# Mother-to-Child Transmission of HTLV-1 Epidemiological Aspects, Mechanisms and Determinants of Mother-to-Child Transmission

**DOI:** 10.3390/v8020040

**Published:** 2016-02-03

**Authors:** Florent Percher, Patricia Jeannin, Sandra Martin-Latil, Antoine Gessain, Philippe V. Afonso, Aurore Vidy-Roche, Pierre-Emmanuel Ceccaldi

**Affiliations:** 1 Pasteur Institute, Virology Department, Epidemiology and Physiopathology of Oncogenic Viruses Unit, F-75015 Paris, France; florent.percher@pasteur.fr (F.P.); patricia.jeannin@pasteur.fr (P.J.); antoine.gessain@pasteur.fr (A.G.); philippe.afonso@pasteur.fr (P.V.A.); aurore.vidy@pasteur.fr (A.V.-R.); 2 UMR CNRS 3569, Paris 75015, France; 3 Sorbonne Paris Cité, Cellule Pasteur, Université Paris Diderot, Institut Pasteur 75015, Paris; 4 ANSES, Enteric Viruses Unit, Maisons-Alfort 94706, France; sandra.martin-latil@anses.fr

**Keywords:** human, HTLV-1, intestinal barrier, retrovirus, breastfeeding

## Abstract

Human T-cell Lymphotropic Virus type 1 (HTLV-1) is a human retrovirus that infects at least 5–10 million people worldwide, and is the etiological agent of a lymphoproliferative malignancy; Adult T-cell Leukemia/Lymphoma (ATLL); and a chronic neuromyelopathy, HTLV-1 Associated Myelopathy/Tropical Spastic Paraparesis (HAM/TSP), as well as other inflammatory diseases such as infective dermatitis and uveitis. Besides sexual intercourse and intravenous transmission, HTLV-1 can also be transmitted from infected mother to child during prolonged breastfeeding. Some characteristics that are linked to mother-to-child transmission (MTCT) of HTLV-1, such as the role of proviral load, antibody titer of the infected mother, and duration of breastfeeding, have been elucidated; however, most of the mechanisms underlying HTLV-1 transmission during breast feeding remain largely unknown, such as the sites of infection and cellular targets as well as the role of milk factors. The present review focuses on the latest findings and current opinions and perspectives on MTCT of HTLV-1.

## 1. Introduction

Human T-cell Leukemia Virus Type 1 (HTLV-1) infects at least 5–10 million people worldwide, mainly in highly endemic areas such as southern Japan, West/Central Africa, the Caribbean region, and parts of South America and Melanesia [1]. HTLV-1 infection is mostly associated with two distinct diseases: a lymphoproliferation, Adult T cell Leukemia/Lymphoma (ATLL), and an inflammatory neurological disease, tropical spastic paraparesis or HTLV-1 associated myelopathy (HAM/TSP). Additionally, HTLV-1 is associated with other inflammatory diseases such as infective dermatitis, some uveitis and some myositis. Although HTLV-1 preferentially infects CD4^+^ T cells [2], CD8^+^ T-cells may play an important role as reservoir in the host [3], and to a lesser extent, infected monocytes and B lymphocytes, dendritic cells, and endothelial cells may be found [4,5]. Different modes of transmission have been identified for HTLV-1: (1) sexual contact; (2) transfusion of contaminated blood; and (3) from mother to child (MTCT) [6]. In each case, such a transmission involves the transfer of infected body fluid (semen, blood, and milk, respectively). In the case of MTCT, cohort studies on HTLV-1 infected carriers indicate that infection during childhood is a potent risk factor for the development of ATLL [7]. It is now clear that HTLV-1 MTCT mainly involves prolonged breastfeeding, as demonstrated by epidemiological, virological and experimental data. However, the mechanisms of such a transmission remain largely unknown. For instance, the nature of the infected cells present in the milk, the anatomical sites of viral entry through the mucosa, the first cellular targets of infection, the role of anti-HTLV-1 antibodies present in breast milk, and the role of other milk factors that may influence MTCT have not been completely addressed. The present review focuses on such mechanisms, current studies and perspectives.

## 2. Evidence of HTLV-1 MTCT during Breastfeeding

First evidence of HTLV-1 transmission from infected mother to children during lactation has been brought by epidemiological studies. HTLV-1 infection was more prevalent among breastfed children than bottle-fed children in Japan [8,9], with a rate of seroconversion of 15.7% among children that had been breastfed for 12 months, compared to 3.6% for bottle-fed children for similar period [10]. Moreover, there is a correlation between MTCT rate and breastfeeding duration. Thus, in a prospective study in Jamaica, Wiktor *et al.* [11] reported that breastfeeding beyond 12 months was associated with a transmission rate of 32%, compared to a transmission rate of 9% for shorter breastfeeding durations. Similarly, Takahashi *et al.* [12] showed that a six-month duration of breastfeeding was a critical point in the rate of seroconversion, since rates of 4.4% and 14.4% were found for children that had been breastfed for periods under six months or over seven months, respectively. A major piece of evidence supporting HTLV-1 transmission through breastfeeding has been brought in the 1980s in Japan, where Hino and coworkers started a pilot study to screen pregnant women in Nagasaki Prefecture for anti-HTLV-1 antibodies (for a review, see [13]). HTLV-1 prevalence was around 4%. Interestingly, HTLV-1 prevalence among the elder children of the HTLV-1 carrier mothers was approximately 20%, and mothers of the HTLV-1 positive children were usually HTLV-1 positive (92%), thus showing evidence of MTCT. More importantly, in 1987, the ATLL Prevention Program Nagasaki, which aimed to refrain seropositive mothers from breastfeeding in the Nagasaki Prefecture, resulted in a huge reduction of HTLV-1 MTCT from 20.3% to 2.5% [14]. The major importance of breastfeeding in HTLV-1 MTCT was later confirmed in other areas [15]. Of note, this residual rate (2.5%) of MTCT in the absence of breastfeeding raised the possibility of minor secondary routes, such as contamination during delivery, or intrauterine transmission. This latter route remains controversial, since contradictory studies on the presence of HTLV-1 in cord-blood samples from seropositive babies have been reported [16,17,18].

From a virological point of view, it is known that many retroviruses may be transmitted via breast milk, such as Moloney murine leukemia virus [19,20], Mouse Mammary Tumor Virus [21], or Caprine Arthritis Encephalitis Virus [22]. Concerning HTLV-1, viral antigens [23], and antibodies to HTLV-1 were found in the milk of seropositive mothers [24]. The proviral load in breast milk is strongly predictive of the risk of MTCT, increasing from 4.7/1000 person-months for a provirus load in milk lower than 0.18% to 28.7/1000 person-months for a provirus load higher than 1.5% [25].

From an experimental point of view, oral inoculation of peripheral blood lymphocytes isolated from ATLL patients to adult common marmosets (*Callithrix jacu*s) was able to induce seroconversion within 2.5 months, and the virus was detected in the animal peripheral blood lymphocytes [26]. This study also demonstrated that 5.6 × 10^7^ cells from ATLL patients were sufficient to infect these animals. Similarly, oral inoculation of concentrated fresh milk from HTLV-1 seropositive mothers in the same animal model could transmit the infection [27]. Oral transmission of HTLV-1 could be likewise observed in other animal models. Oral inoculation of four rabbits for eight weeks with an HTLV-1-infected rabbit lymphoid cell line resulted in the seroconversion of one animal, and it was possible to generate from this animal a lymphoid cell line productively infected with HTLV-1 [28]. Similarly, oral inoculation of HTLV-1-infected lymphoid cell line (*i.e.*, MT-2) to rats induced a persistent HTLV-1 infection in the absence of both humoral and cellular immune responses [29].

## 3. The Mechanisms of HTLV-1 MTCT

Altogether, these data indicate that breastfeeding is a major route for HTLV-1 MTCT. However, the mechanisms of HTLV-1 passage through the digestive tract remain largely unknown.

A first point to address concerns the source of HTLV-1 infection in breast milk. It is known that cell-to-cell contact is required for efficient viral transmission *in vivo* [30] as well as *in vitro* [31,32], except for dendritic cells that can be infected directly with cell-free HTLV-1 virions [33]. Cell free virions have not been detected so far in breast milk, thus the potential source of infection in breast milk may come from infected cells, such as lymphocytes, macrophages, or breast epithelial mammary cells. Since it has been estimated that breastfed children ingest an average of 10^8^ leucocytes a day, considering prolonged breastfeeding [34,35], infected lymphocytes could provide a strong source of infection in milk [36]. HTLV-1 infected mononuclear cells can be found in the milk from seropositive mothers during early lactation [23,37]. Of note, cellular components in breast milk can be found even after long-term lactation (over 5 years) [38], even if the ratio between the different cell types varies along the time: for example, the major part of cells in mother’s early milk and colostrum is constituted of macrophages [39]. It has been found that leukocytes and epithelial cells from the mammary gland are susceptible to HTLV-1 infection [38]. This observation was confirmed by the evidence that mammary basal epithelial cells can be productively infected with HTLV-1 and are able to transfer infection to peripheral blood lymphocytes [40,41]. In addition, in a case report of an ATLL male patient with pseudogynecomasty, breast biopsy revealed the presence of mammary epithelial cells productively infected with HTLV-1 [42]. Such data support the hypothesis that basal and/or luminal epithelial cells may constitute a reservoir of HTLV-1 infectivity. The importance of mammary epithelial cells in viral transmission during lactation has also been evoked for Bovine Leukemia Virus (BLV), another deltaretrovirus that is transmitted from BLV-infected cows to calves during lactation [43]. Whatever the cell types involved, this can provide a more or less continuous source of infection.

A second question concerning the mechanisms of HTLV-1 transmission through the digestive tract is the anatomical site of viral entry. Up until now, no studies have addressed this question; in fact, animal models studies (marmoset, rabbit, and rat) on oral inoculation of HTLV-1 were mainly focused on seroconversion, progression towards an ATLL-like disease, and immune status of the host. Among the different possible sites of entry, the palatine tonsils and gut seem to be of particular importance due to their enrichment in possible targets (lymphoid cells and M cells), their function in antigen sampling [44] and their structure. Moreover, although it does not constitute a proof of viral entry, HTLV-1 may be retrieved in these structures, as shown in the tonsils for HTLV-1 infected humans [45], in the intestine and mesenteric lymph nodes of squirrel monkeys inoculated intravenously with HTLV-1 [46] and in Gut-Associated-Lymphoid Tissues from orally inoculated rabbits [47]. Whatever the primary sites of HTLV-1 passage/infection, tonsils and/or gut, the virus encounters an epithelium, pluristratified or monostratified, respectively. This allows *in vitro* studies using classical models of human epithelial cell monolayers.

**Figure 1:**
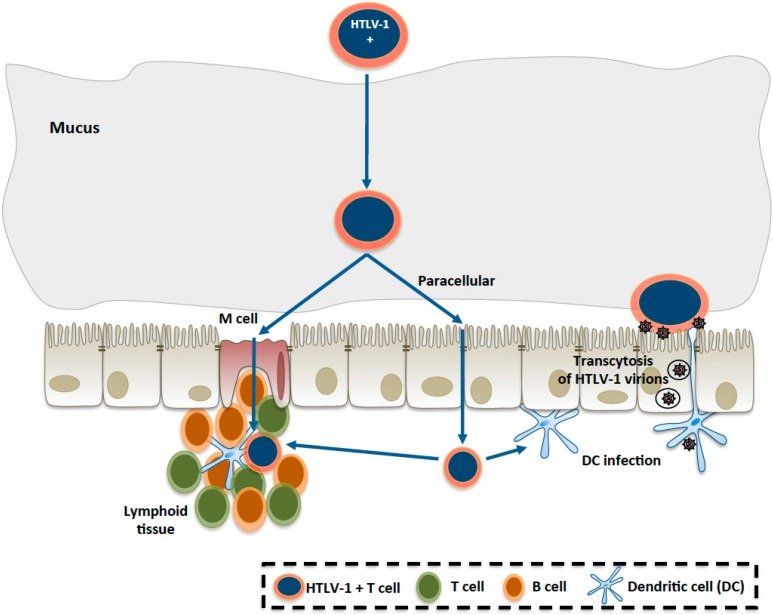
Hypothetical mechanisms of HTLV-1 (Human T-cell Lymphotropic Virus type 1 ) passage through the intestinal epithelium.

Very few *in vitro* studies have been published concerning the mechanisms of passage of HTLV-1 across the intestinal barrier. In 1992, Zacharopoulos *et al.* [48] indicated that a human enterocytic cell lines (*i.e.*, I407) was susceptible to HTLV-1 infection *in vitro*, as shown by electron microscopy, *in situ* hybridization, and PCR amplification. However, it was unclear if the cells were fully differentiated as no data on the epithelium integrity and tight junctions were shown. In contrast, our laboratory performed studies on the susceptibility of three different human enterocytic cell lines on compartmentalized culture devices, with assessment of the enterocyte differentiation: existence of a tight epithelial barrier was checked by electron and confocal microscopy, and the trans-epithelial resistance was measured [49]. In this study, as summarized in Figure 1, it was demonstrated that HTLV-1 infected lymphocytes were unable to disrupt the epithelial barrier integrity or infect human enterocytes, in contrast to previous studies on a blood–brain barrier model in human brain endothelial cells [5,50]. However, it was shown that HTLV-1 virions were able to cross the epithelial barrier by transcytosis mechanism, and productively infect underlying human dendritic cells [49].

HTLV-1-infected lymphocytes, once they have crossed the mucus layer, could either pass through the epithelium via M cells, or in-between enterocytes (paracellular passage), to reach the lamina propria where potential targets of HTLV-1 infection, such as T lymphocytes (and/or dendritic cells and B lymphocytes) are located. From *in vitro* studies [49], the infection of enterocytes or the epithelial layer disruption seems to be excluded. Another possibility, suggested in the same study, could be viral transcytosis through the enterocyte, with infection of underlying dendritic cells.

Interestingly, viral transcytosis through enterocytes was observed only in the presence of HTLV-1 infected lymphocytes, and not in the case of purified virions. Such a mechanism of viral transcytosis is reminiscent of previous work showing transcytosis of HIV across an epithelial barrier, without infection of enterocytes, and subsequent infection of macrophages or CD4 lymphocytes located to the basal side of the epithelium [51]. These *in vitro* results highlight one of the potential mechanisms proposed for HTLV-1 passage, which are summarized in Figure 1.

## 4. Determinants of HTLV-1 MTCT

Studies on HTLV-1 MTCT determinants have focused mainly on genetic host factors, immunological host factors, lactation duration and milk components.

The genetic host factors that control HTLV-1 infection by breastfeeding have been investigated by Plancoulaine *et al.* [52], who began in the 1990s a large epidemiological study in endemic villages of French Guiana. The authors found a dominant major gene predisposing to HTLV-1 infection, in addition to the expected familial correlations due to the transmission routes (mother to child and spouse to spouse) [53]. Previous studies had shown that *HLA* (Human Leukocyte Antigen) genes distribution was different for ATLL or HAM/TSP patients compared to asymptomatic carriers (for example, see [54]), but the study by Plancoulaine *et al.* [53] indicated a genetic predisposition for HTLV-1 infection itself for 1.5% of the population, which concerned almost all infected children under 10 years of age, *i.e.*, infected through breastfeeding. Further studies allowed mapping a major susceptibility locus for HTLV-1 infection during childhood to chromosome 6q27 [55].

Concerning the immunological factors involved in HTLV-1 MTCT, the role of maternal anti-HTLV-1 antibodies may appear controversial. Such studies have to take into account the duration of breastfeeding, since the protective role of anti-HTLV-1 antibodies has been demonstrated in a rabbit model of infection, where passive immunization was shown to prevent milk-borne transmission of HTLV-1 to offspring [56]. Moreover, it has been shown *in vitro* that the addition of HTLV-1 serum cord blood plasma is able to prevent the infection of human neonatal lymphocytes when co-cultured with breast-milk cells of HTLV-1 carrier mothers [12]. However, it has been suggested that higher anti-HTLV-1 antibodies titer in the serum of the mother, as well as the presence of anti-Tax antibodies, is associated with a higher risk of children infection [11,52,57,58,59]. However, a high anti-HTLV-1 antibody titer in the serum may be correlated with a high provirus load in PBMCs, which is a risk factor for HTLV-1 MTCT [57]. In an analysis including the provirus load in maternal PBMCs, the presence of anti-Tax antibodies and the anti-HTLV-1 titers, it was found that a higher maternal proviral load and a higher anti-HTLV-1 antibody titer were independently associated with a higher risk of HTLV-1 MTCT, whereas the presence of anti-Tax antibodies was not [60].

Another point to take into account concerns the other milk components that may influence HTLV-1 MTCT transmission. As an example, it has been shown that lactoferrin, an iron-binding milk glycoprotein, was able to enhance HTLV-1 replication, by transcriptional activation of HTLV-1 Long Terminal Repeat (LTR), the viral promoter [61]. This effect on HTLV-1 infection seems to be specific since lactoferrin did not show any effect on HIV-1 LTR, and was even able to inhibit HIV-1 infection, probably by interfering with viral fusion and entry steps [61]. As an interesting example of “positive feedback”, the same authors further demonstrated that lactoferrin expression was up-regulated during HTLV-1 infection, probably in a paracrine manner involving Tax-induced NF-κB activation [62].

## 5. Ongoing Research on HTLV-1 MTCT and Perspectives

One of the major remaining questions on MTCT concerns the sites of primary passage/infection of HTLV-1 in the digestive tract. The mechanisms of HTLV-1 infection after oral inoculation should be addressed *in vivo* using a humanized mouse as a model of HTLV-1 infection [63,64,65], in complement to the rabbit model. In particular, combination of histopathological studies and bioluminescence imaging will allow determining the preferential sites of HTLV-1 entry (palatine tonsils, and/or gut). In parallel, the use of transgenic, knock-out, and knock-in mice depleted for different cell types (M cells, dendritic cells, and macrophages) will allow assessing the role of the different cell types in the first steps of infection. These studies will also benefit from an *in vitro* approach, such as differentiation of M cells from enterocytic cell lines, on compartmentalized culture devices, as already done to show the role of these cells in virus transport across the epithelium [66]. Combination of *in vivo*/*in vitro* studies will also allow delineating the role of factors such as milk components (e.g., lactoperoxidase), the proviral load, and the antibody titer in HTLV-1 transport through the intestinal epithelium.

Another perspective concerns the comprehension of the role of breastfeeding duration on HTLV-1 MTCT. It seems rather clear that such a role corresponds to a combination of the cumulative viral input, the changes over time in milk composition in infected cell types, maternal antibodies, and the immune status and maturation of the neonate’s gut. It is known that neonatal life (as well as prenatal) organizes and controls mucosal homeostasis through endogenous and exogenous factors that drive the development and maturation of the intestinal immune system (for a review, see [67]). Recent studies have shown the effects of intestinal microbiota in the development of the immune system and intestinal architecture [68,69]. Gut microbiota could have an impact on HTLV-1 MTCT efficiency, as reported for HIV-1 [70] or Mouse Mammary Tumor Virus [71], as well as the human milk microbiota, which participate in the neonate’s gut microbiota constitution [72].

Altogether, these further studies could delineate new preventive strategies to counteract HTLV-1 MTCT, as well as provide new data on the general mechanisms of pathogenic agents in MTCT.
